# Supplementary figures and images for: Interferon-γ responses to Plasmodium falciparum vaccine candidate antigens decrease in the absence of malaria transmission
Source: PeerJ. 2017 Jan 10;5:e2855. doi: 10.7717/peerj.2855 (PMC5228499; doi:10.7717/peerj.2855)

## A. April 2008

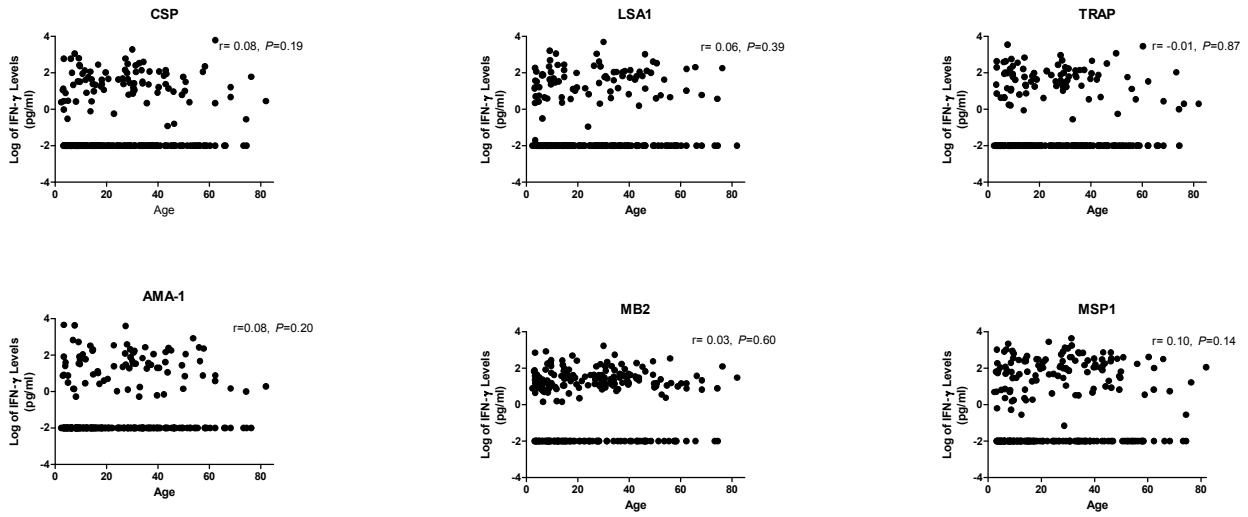

## B. October 2008

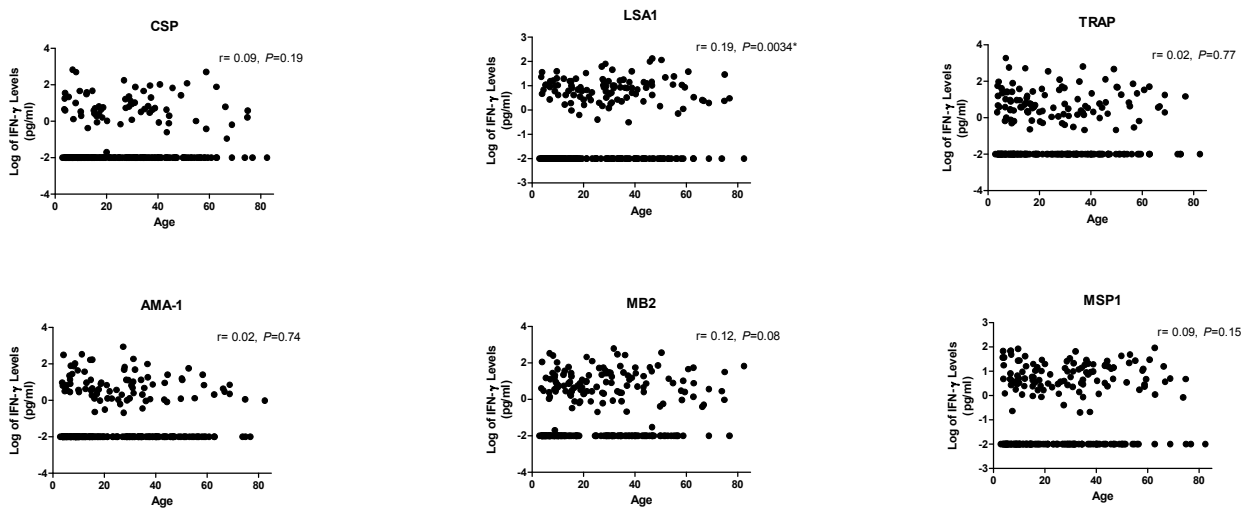

## C. April 2009

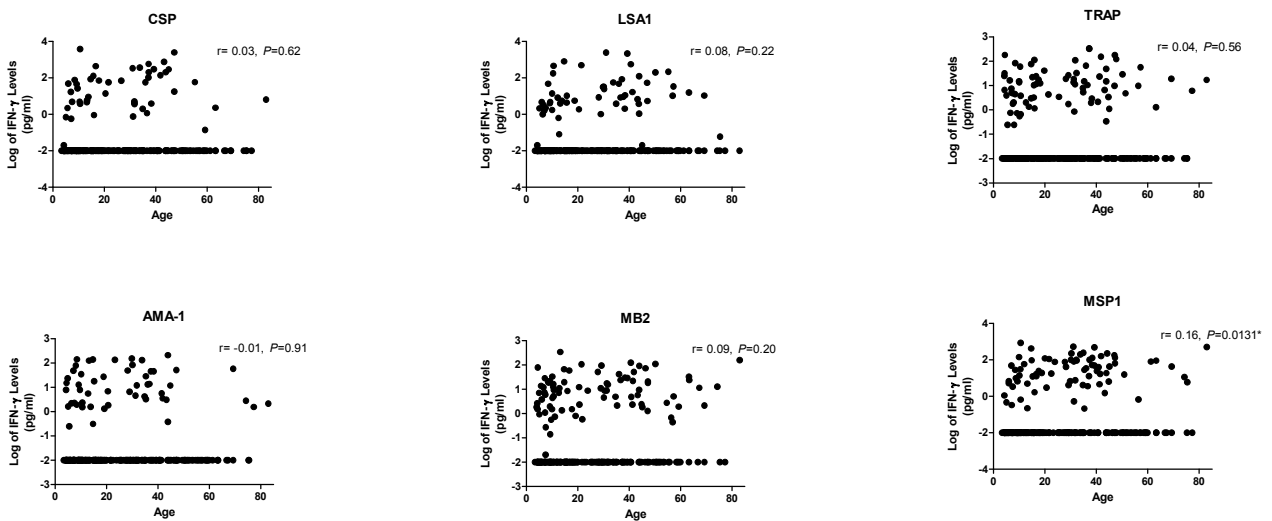

Supplement: Figure S1 [file peerj-05-2855-s001.pdf]
