# Supplementary material for: Interferon-γ responses to Plasmodium falciparum vaccine candidate antigens decrease in the absence of malaria transmission
Source: PeerJ. 2017 Jan 10;5:e2855. doi: 10.7717/peerj.2855 (PMC5228499; doi:10.7717/peerj.2855)
Supplement: Table S1 [file peerj-05-2855-s002.docx]

Supplementary Table 1. Antibody prevalence and levels to circumsporozoite protein (CSP) and schizont extract

| *P. falciparum* Antigen | Antibody Prevalence (%) | | | Median (25^th^ -75^th^ Percentile) Antibody Levels | | |
| --- | --- | --- | --- | --- | --- | --- |
|  | April 2008 | April 2009 | *P*^a^ | April 2008 | April 2009 | *P*^b^ |
| CSP | 8.3 | 4.5 | 0.11 | 0.6 (0.4 – 0.8) | 0.5 (0.4 – 0.6) | <0.0001 |
| schizont extract | 7.0 | 0.0 | <0.0001 | 0.5 (0.4 – 0.7) | 0.4 (0.3 – 0.4) | <0.0001 |

^a^ Prevalence of antibodies between April 2008 and April 2009 were compared by McNemar’s test

^b^ Levels of antibodies between April 2008 and April 2009 were compared by Wilcoxon-signed rank sum test
