# Supplementary material for: Interferon-γ responses to Plasmodium falciparum vaccine candidate antigens decrease in the absence of malaria transmission
Source: PeerJ. 2017 Jan 10;5:e2855. doi: 10.7717/peerj.2855 (PMC5228499; doi:10.7717/peerj.2855)
Supplement: Table S2 — IFN-γ levels to P. falciparum antigens CSP, LSA-1, MB2, TRAP, AMA-1, MB2 and MSP-1 in 30 samples collected in April 2008, October 2008 and April 2009. [file peerj-05-2855-s003.docx]

Supplementary Table 2. Batch analysis of IFN-γ levels to *P. falciparum* antigens in 30 samples collected in April 2008, October 2008 and April 2009.

| Antigen | Median (10^th^ , 90^th^ percentile) pg/ml | | |  |
| --- | --- | --- | --- | --- |
|  | April 2008 | October 2008 | April 2009 | *P^a^* |
| CSP | 192.4 (0.0, 850.1) | 0.0 (0.0, 842.8) | 10.64 (0.0, 134.2) | < 0.0001 |
| LSA-1 | 168.5 (0.15, 1722) | 0.0 (0.0, 45.32) | 0.0 (0.0, 179.4) | < 0.0001 |
| TRAP | 51.46 (0.0, 496.8) | 0.0 (0.0, 110.8) | 0.0 (0.0, 6.16) | 0.0001 |
| AMA-1 | 0.0 (0.0, 487.5) | 0.0 (0.0, 27.06) | 0.0 (0.0, 24.02) | 0.0576 |
| MB2 | 0.0 (0.0, 361.3) | 0.0 (0.0, 6.156) | 0.0 (0.0, 23.48) | 0.0024 |
| MSP-1 | 242.2 (0.0, 1160) | 0.0 (0.0, 12.71) | 0.0 (0.0, 36.46) | < 0.0001 |

^a^ Differences in IFN-γ levels over time were compared by Kruskal-Wallis test.
